# Genome-wide identification of functional enhancers and their potential roles in pig breeding

**DOI:** 10.1186/s40104-022-00726-y

**Published:** 2022-07-04

**Authors:** Yinqiao Wu, Yuedong Zhang, Hang Liu, Yun Gao, Yuyan Liu, Ling Chen, Lu Liu, David M. Irwin, Chunhui Hou, Zhongyin Zhou, Yaping Zhang

**Affiliations:** 1 State Key Laboratory of Genetic Resources and Evolution, and Yunnan Laboratory of Molecular Biology of Domestic Animals, Kunming Institute of Zoology, Chinese Academy of Sciences, Kunming, 650223 Yunnan China; 2 Kunming College of Life Science, University of Chinese Academy of Sciences, Kunming, 650204 Yunnan China; 3 State Key Laboratory for Conservation and Utilization of Bio-resource in Yunnan, Yunnan University, Kunming, 650091 Yunnan China; 4 School of Life Science, Yunnan University, Kunming, 650091 Yunnan China; 5 Institute of Physical Science and Information Technology, Anhui University, Hefei, 230601 Anhui China; 6 Department of Laboratory Medicine and Pathobiology, University of Toronto, Toronto, Canada; 7 Department of Biology, School of Life Sciences, Southern University of Science and Technology, Shenzhen, 518055 China; 8 Center for Excellence in Animal Evolution and Genetics, Chinese Academy of Sciences, Kunming, 650223 Yunnan China

**Keywords:** Breeding, Functional enhancers, Pig, STARR-seq

## Abstract

**Background:**

The pig is an economically important livestock species and is a widely applied large animal model in medical research. Enhancers are critical regulatory elements that have fundamental functions in evolution, development and disease. Genome-wide quantification of functional enhancers in the pig is needed.

**Results:**

We performed self-transcribing active regulatory region sequencing (STARR-seq) in the porcine kidney epithelial PK15 and testicular ST cell lines, and reliably identified 2576 functional enhancers. Most of these enhancers were located in repetitive sequences and were enriched within silent and lowly expressed genes. Enhancers poorly overlapped with chromatin accessibility regions and were highly enriched in chromatin with the repressive histone modification H3K9me3, which is different from predicted pig enhancers detected using ChIP-seq for H3K27ac or/and H3K4me1 modified histones. This suggests that most pig enhancers identified with STARR-seq are endogenously repressed at the chromatin level and may function during cell type-specific development or at specific developmental stages. Additionally, the *PPP3CA* gene is associated with the loin muscle area trait and the *QKI* gene is associated with alkaline phosphatase activity that may be regulated by distal functional enhancers.

**Conclusions:**

In summary, we generated the first functional enhancer map in PK15 and ST cells for the pig genome and highlight its potential roles in pig breeding.

**Supplementary Information:**

The online version contains supplementary material available at 10.1186/s40104-022-00726-y.

## Background

A fundamental goal in modern biology is to identify and characterize non-coding regulatory elements that control gene expression in development and disease [1, 2]. Enhancers, as genomic non-coding sequences, are critical regulatory elements that control spatial and temporal gene expression [3]. Variation at enhancers is associated with complex traits and diseases [4, 5]. Previous studies found that enhancers have crucial roles in sexual development in mammals and that duplication or deletion of some of these enhancers could result in sex reversal [6, 7]. ZRS is a well-characterized long-range enhancer directing limb-specific sonic hedgehog (*Shh*) expression. Kvon et al. [8] employed a combination of comparative genomics and genetic engineering to introduce snake-specific deletions into the orthologous genomic region of the ZRS enhancer in the mouse that resulted in severe limb reduction. Thus, it is important to identify enhancers and reveal their biological functions.

The domestic pig (*Sus scrofa*) is an economically important livestock species and is widely used as a large animal model for preclinical studies due to its human-like size and physiology [9, 10]. A number of studies performed in the pig, based on sequence homology information [11] and chromatin marks, including DNA accessibility, histone modification and transcription factor or cofactor binding, have been conducted to characterize regulatory elements such as enhancers [12–14]. However, these currently used predictive methods do not provide direct functional evidence or quantify the enhancer activity. More recently, the development of the self-transcribing active regulatory region sequencing (STARR-seq) strategy has allowed direct measurement of enhancer activity across the whole genome [15]. This method was first applied in *Drosophila* [15, 16] and later in human cells [17–19] and other species [20–23]. However, to date, no genome-wide studies have been performed to detect functional enhancers in the pig genome.

Here, we performed STARR-seq in the porcine kidney epithelial PK15 and testicular ST cell lines to generate the first whole genome-wide enhancer activity quantification map for the pig. We identified 2576 functional enhancers, most of which are located in repetitive sequences and enriched within silent and lowly expressed genes. We compared our map to ATAC-seq and ChIP-seq data and found that our enhancers poorly overlap with accessible chromatin regions and were highly enriched in chromatin with the repressive histone modification H3K9me3. Moreover, mapping of enhancers to complex traits in the pig found that the *PPP3CA* gene, which is associated with the loin muscle area trait, and the *QKI* gene, which is associated with alkaline phosphatase activity, might be regulated by distal enhancers. In summary, we provide the first functional enhancer map for the pig that should promote the identification of causal mutations for complex traits in the pig.

## Methods

### Cell culture

Pig (*Sus scrofa*) PK15 cells were maintained in DMEM media (Gibco, Thermo Fisher Scientific, Shanghai, China) and ST cells were maintained in MEM media (Gibco, Thermo Fisher Scientific, Shanghai, China) supplied with 10% FBS (Gibco, Thermo Fisher Scientific, Shanghai, China) and 1% penicillin/streptomycin (Thermo Fisher Scientific, Shanghai, China). All cells were tested for mycoplasma using Myco-Blue Mycoplasma Detector (Vazyme, Nanjing, China).

### Construction of STARR-seq input plasmid libraries

STARR-seq input plasmid libraries were generated as described in a previous study [15]. Briefly, genomic DNA was extracted from an ear tissue sample from one Diannan small-ear pig. After sonication with a Scientz-II D ultrasonic generator (NingBo Scientz Biotechnology, Ningbo, China), 500–800 bp sheared DNA was selected by cutting from a gel and was recovered using E.Z.N.A.® Gel Extraction Kit (Omega Bio-Tek, Norcross, GA, USA). About 6 μg of DNA fragments were end repaired, 5′-phosphorylated, 3'dA-tailed and ligated with Illumina adaptor using the VAHTS Mate Pair Library Prep Kit for Illumina® (Vazyme, Nanjing, China) following the manufacturer’s protocol. Ligated DNA was amplified with TransStart FastPfu Fly DNA Polymerase (Transgen, Beijing, China) with homology arms primers (Forward primer: 5'-ACA CTC TTT CCC TAC ACG ACG-3' and Reverse primer: 5'-GAC TGG AGT TCA GAC GTG TGC-3'). The vector backbone was modified from pGL3-basic (Promega, Beijing, China). The Super Core Promoter 1 (SCP1) promoter was inserted upstream of the luciferase site. The luciferase sequence was replaced with a GFP sequence containing a synthetic intron and homology arms. The homology arms were used to insert the genomic DNA fragments. The vector backbone was linearized using PCR (95 °C for 5 min; then 25 cycles of 95 °C for 20 s, 57 °C for 20 s and 72 °C for 4 min; Forward primer: 5'-CGT CGT GTA GGG AAA GAG TGT-3' and Reverse primer: 5'-GCA CAC GTC TGA ACT CCA GTC-3'). Further, circular plasmids were removed using DpnI (New England BioLabs, Ipswich, MA, USA) at 37 °C for 30 min. Linearized vector was separated using a 1% agarose gel and purified with E.Z.N.A.® Gel Extraction Kit. We then recombined the genomic DNA fragments into the linearized vector using ClonExprress II One Step Cloning Kit (Vazyme, Nanjing, China). The 10 μL of the recombined plasmids transformed into Trans1-T1 Phage Resistant Chemically Competent Cell (Transgen, Beijing, China). A total of 140 transformation reactions were pooled together in 4 L LB medium with 10 μg/mL Ampicillin, which was grown to an Optical Density (OD) of 0.8. Plasmids were purified using E.Z.N.A.® Endo-Free Plasmid Maxi Kit (Omega Bio-Tek, Norcross, GA, USA).

### Transfection of STARR-seq input plasmid libraries into cells

The STARR-seq input plasmid library was transfected into pig PK15 and ST cells using TurboFect™ Transfection Reagent (Thermo Fisher Scientific, Waltham, MA, USA) according to the manufacturer’s protocol. The inhibitors BX-795 (MedChemExpress, Shanghai, China) and C16 (Sigma Aldrich, Shanghai, China) were used to suppress the expression of immune-reated genes in transfected PK15 and ST cells. For PK15 cells, 1 μmol/L of both inibitors was added to the culture medium, while for ST cells, 0.5 μmol/L of both inhibitors was used. We generated two biological replicates of the PK15 and ST cells.

### Construction of cDNA and input plasmid libraries for Illumina sequencing

Total RNA was extracted from the transfected PK15 and ST cells using the TransZol™ Up Plus RNA Kit (Transgen, Beijing, China). The poly(A)^+^ RNA fraction was enriched using VAHTS mRNA Capture Beads (Transgen, Beijing, China). Genomic DNA was digested with 5 U DNase I (New England BioLabs, Ipswich, MA, USA) at 37 °C for 20 min. First strand cDNA was synthesized using TransScript One-Step gDNA Removal and cDNA synthesis SuperMix kit (Transgen, Beijing, China) at 50 °C for 30 min and 85 °C for 5 s with the library specific primer (5'-GAC TGG AGT TCA GAC GTG TGC-3'). We used 10–30 ng cDNA as template in a 50-μL PCR reaction with TransStart FastPfu Fly DNA Polymerase with the reporter gene specific primers (Forward primer: 5'-AAC AAG AAT TGG GAC AAC TCC AGT GAA-3' and Reverse primer: 5'-GAC TGG AGT TCA GAC GTG TGC-3'). PCR products were purified by GeneJET PCR Purification Kit (Thermo Fisher Scientific, Waltham, MA, USA). Purified PCR products were used as templates for the second round PCR (98 °C for 2 min; followed by 6–10 cycles of 98 °C for 30 s, 65 °C for 30 s, 72 °C for 30 s; and then 72 °C for 5 min) with TransStart FastPfu Fly DNA Polymerase and the index primers in the VAHTS Multiplex Oligos set1/set2 for Illumina Kit (Vazyme, Nanjing, China). Second round PCR products were then purified using a GeneJET PCR Purification Kit.

After capture of poly(A)^+^ RNA, the remaining aqueous solution was treated with 10 μL RNase A (Transgen, Beijing, China) at 37 °C for 60 min to remove any RNA in the solution. Input plasmid templates were purified with a GeneJET PCR Purification Kit. Purified plasmid DNA was amplified with the TransStart FastPfu Fly DNA Polymerase and the index primers from the VAHTS Multiplex Oligos set1/set2 for Illumina.

Both the cDNA and input plasmid libraries were sequenced on the Illumina HiSeq X Ten platform at BerryGenomics (Beijing, China).

### Reporter assay

To verify the enhancers identified in this study, we used the input plasmid library backbone and inserted candidate regions. Plasmids were co-transfected with luciferase control vector (pGL3-promoter (Promega, Beijing, China)) into PK15 cells with TurboFect™ Transfection Reagent following the manufacturer’s protocol. Primers of candidate regions are reported in Additional file 1: Table S1.

### RT-qPCR

RNAs were extracted using TransZol™ Up Plus RNA Kit. Contaminating genomic DNA was digested with 5 U DNase I and cDNAs was then synthesized using the HiScript III 1st Strand cDNA Synthesis Kit (Vazyme, Nanjing, China). RT-qPCR used ChamQ Universal SYBR qPCR Master Mix (Vazyme, Nanjing, China) with 95 °C for 5 min, followed by 40 cycles of 95 °C for 15 s, 60 °C for 30 s with the melting curve program. Primers used for RT-qPCR are described in Additional file 2: Table S2.

### Computational processing of STARR-seq data

To remove low-quality reads and adaptor sequences, the STARR-seq data was processed using Trimmomatic v0.39 with following parameters: TruSeq2-PE.fa:2:30:10 LEADING:3 TRAILING:3 SLIDINGWINDOW:4:15 MINLEN:36 [24]. Reads were then aligned to the pig reference genome (Sscrofa11.1) using Bowtie2 v2.3.5.1 with parameter “--no-discordant -X 2000” [25]. Mapped reads were filtered using SAMtools v1.3.1 with parameter “view -bS -f 2 -q 5” to obtain uniquely mapped reads [26]. PCR duplicates were removed using the Picard toolkit [27]. Reproducibility of the two independent biological replicates in PK15 and ST cells were evaluated using Pearson correlation coefficients, which were calculated using multiBamSummary and plotCorrelation in deepTools v3.5.1 [28]. Enhancers were identified using BasicSTARRseq as described in previous studies [15, 21] with a strength > 1.0, *P* value < 0.001 and FDR < 0.1.

### RNA-seq library construction and analysis

RNA was extracted from PK15 cells using the TransZol™ Up Plus RNA Kit. Libraries construction and sequencing were performed at Novogene.

Trimmomatic v0.39 [24] was used to filter low-quality reads with default parameter. Reads were aligned to the pig genome (Sscrofa11.1) using HISAT2 v2.2.1 [29]. The expression values of each gene was calculated using StringTie v2.1.4 [30].

### Potential biological function analysis

To obtain potential biological functions of the enhancers identified in this study, each enhancer was assigned to a putative gene based on the closest genomic distance. Gene Ontology (GO) enrichment analysis of the putative genes was performed using DAVID [31]. In addition, we identified potential transcription factor binding sites in the putative enhancers using the MEME-ChIP web server [32].

### ATAC-seq library construction and analysis

ATAC-seq library preparation was performed as previously described [33]. PK15 cell nuclei (~ 100,000) were extracted using lysis buffer (10 mmol/L Tris-HCl (pH 7.4), 10 mmol/L NaCl, 3 mmol/L MgCl_2_, 0.1% IGEPAL CA-630) and incubated with Tn5 transposase (Vazyme, Nanjing, China). Next, the transposed DNA was amplified through 14 PCR cycles with indexed primers according to the manufacturer’s protocol for the TruePrep Index Kit V4 for Illumina (Vazyme, Nanjing, China). We sequenced two independent libraries using the Hiseq X ten platform (BerryGenomics, Beijing, China).

Trimmomatic 0.39 [24] with default parameter was used to filter low-quality reads and adaptor sequences. Reads were then aligned to the pig reference genome (Sscrofa11.1) using BWA-MEM v0.7.17 [34]. Mapped reads were processed with SAMtools v1.3.1 [26] to keep uniquely mapped reads. PCR duplicates were removed using the Picard toolkit [27]. Effective reads were generated by removing mitochondrial reads using BEDTools v2.25.0 [35]. Peaks were called using MACS v2.1.0 (setting: -f BAMPE -q 0.001 --shift − 75 --extsize 150 --nomodel -B --SPMR -g 2.5e9) [36].

### ChIP-seq library construction and analysis

ChIP-Seq libraries were prepared following the ENCODE guidelines [37]. ChIP-seq data was mapped to the pig genome (Sscrofa11.1) using Bowtie2 v2.3.5.1 [25]. MACS v2.1.0 [36] was used to identify peaks (default settings for CTCF, H3K9me3; broad peaks model for H3K4me1, H3K4me3, H3K27ac and H3K27me3) with *P* values less than 0.001.

### Hi-C analysis

We identified TADs and Hi-C contacts from the Hi-C data, which was download from the SRA database (accessions number: PRJEB40576 [38] and PRJNA482496 [39]). We then aligned the reads to the pig genome (Sscrofa11.1) using BWA-MEM v0.7.17 [34] and applied HiCExplorer v3.4.2 [40] to build Hi-C contact matrixes with 40 Kb resolution. We identified TADs using HiCExplorer v3.4.2 [40].

## Results

### Quantifying genome-wide enhancer activity in the pig using STARR-seq

**Fig. 1:**
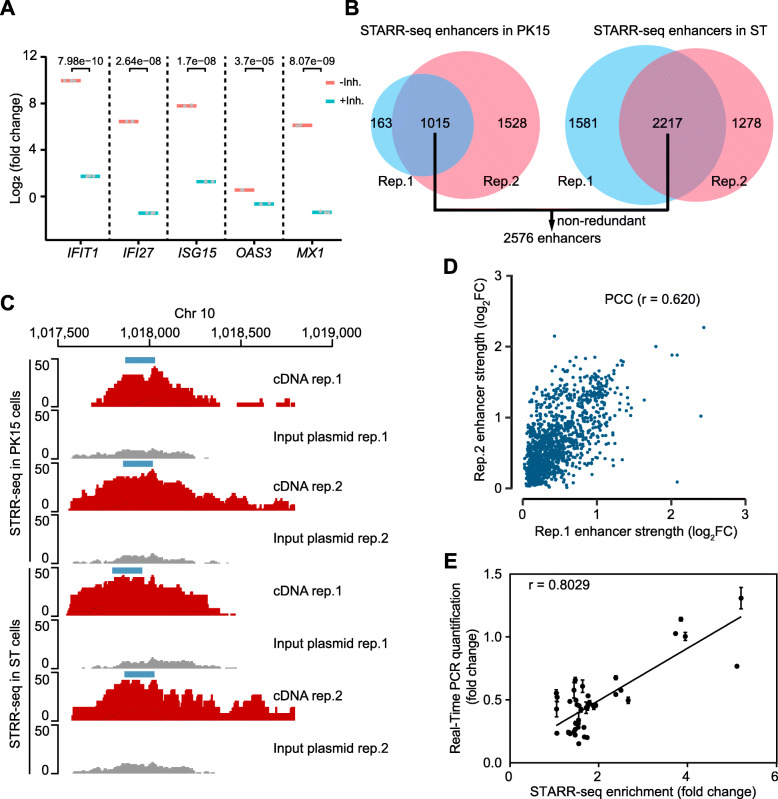
Genome-wide quantification of pig enhancer activity using STARR-seq. **A** Assessment of the immunoreaction and treatment of TBK1/IKK/PKR inhibitors after DNA was transfected into PK15 cells. Expression levels were assessed by RT-qPCR and normalized to non-transfected cells. Bars represent mean fold change across three independent replicates (grey dots). *P*-values were calculated from a *t*-test. **B** Statistics of functional enhancers identified in PK15 and ST cells. Venn diagram shows that enhancers overlap in the two biological replicates. **C** STARR-seq cDNA (red) and input plasmid (gray) fragment densities at representative pig genomic loci. Blue boxes denoted the identified enhancers in the PK15 and ST cells. **D** Correlation analysis of enhancer strength in the two biological replicates of PK15 cells. The correlation was evaluated using the Pearson’s correlation coefficient (PCC). Enhancer strength was calculated based on fold change (FC, cDNA read counts divided by input plasmid read numbers) using 600 bp windows along each chromosome. **E** STARR-seq enhancer enrichment and RT-qPCR quantification of GFP gene expression was linearly correlated. r, Pearson correlation coefficient; Error bars indicate two independent biological replicates

To comprehensively identify putative enhancers with activity in the pig genome, we constructed libraries of randomly fragmented genomic DNA from one Diannan small-ear pig. Furthermore, we used porcine PK15 and ST cells to perform STARR-seq to quantify the activity of the enhancers. Since transfection of most mammalian cells with a plasmid DNA causes a striking increase in the expression of immune-related genes, through the activation of their enhancers, leading to false positive signals with STARR-seq methods [18], thus, we first assessed the immunoreaction of PK15 and ST cells after plasmid transfection. As expected, we found that immune-related genes were highly expressed after transfection with plasmids (Fig. 1A and Additional file 3: Figs. S1 and S2). Therefore, we treated these cell lines with the TBK1/IKKe inhibitor BX-79521 and the PKR inhibitor C1622 during plasmid transfection to decrease expression of immune system-related genes (Fig. 1A and Additional file 3: Figs. S1 and S2). We found that 1 μmol/L of both BX-7951 and C1662 inhibitors in PK15 cells and 0.5 μmol/L of both BX-7951 and C1662 in ST cells reduced the expression of immune system-related genes (Fig. 1A and Additional file 3: Figs. S1 and S2).

The STARR-seq libraries generated from these two cell lines contained between 43.7 and 46.0 million unique fragments in the input plasmid libraries and from 11.9 to 21.0 million unique fragments in their cDNA libraries (Additional file 4: Table S3). The median fragment length of the input plasmid and cDNA libraries were about 600 bp (Additional file 3: Fig. S3). cDNA and input plasmid libraries covered between 78.2% and 92.5% of the non-repetitive sequence of the pig genome (Additional file 3: Fig. S4). The GC-content for each library showed that the sequencing libraries were unbiased (Additional file 3: Fig. S5). Correlation analysis of the two biological replicates of the plasmid and cDNA libraries showed high correlation for both PK15 and ST cells (Additional file 3: Fig. S6A and B). From the above, these results indicated that STARR-seq could be used to quantify pig enhancers activity at a genome-wide scale.

We calculated the enrichment of cDNA reads normalized by input plasmid for 600 bp bins across the whole genome. Potential enhancers were identified as described [15, 21] using BasicSTARRseq with a binomial test *P* < 0.001, enrichment > 1.0, FDR < 0.1. Between 1015 to 2217 enhancers were detected in the pig genome in the PK15 and ST replicates (Fig. 1B and Additional file 5: Table S4). Moreover, we found that 601 of the enhancers (> 50% reciprocal overlap) were shared between the two cell lines (Additional file 5: Table S4). An enhancer located on chromosome 10 is shown in Fig. 1C as an example. The identified enhancers showed a wide range of strengths (Additional file 3: Fig. S6C and D). The Pearson correlation coefficient of the strengths for the two replicates was 0.620 and 0.707 in the PK15 and ST cells, respectively (Fig. 1D and Additional file 3: Fig. S7), demonstrating that the STARR-seq libraries were reproducible in the pig genome. Combining the enhancers found in the PK15 and ST cells resulted in the identification of 2576 non-redundant enhancers in the pig genome (Fig. 1B and Additional file 5: Table S4), which display low overlap with the enhancers identified by ChIP-seq for the H3K27ac or/and H3K4me1 chromatin modifications (Additional file 3: Fig. S8) [12, 14]. To verify the strength of the enhancers identified using STARR-seq, 40 enhancers were selected (Additional file 1: Table S1), with a wide range of strengths, and measured using RT-qPCR. STARR-seq enhancer strength and RT-qPCR of reporter gene expression levels were highly correlated (*r* = 0.8029, ***P < 0.0001, Fig. 1E). These results demonstrate that the enhancers identified in this study are reliable and that pig enhancers identified using histone modifications need to be verified using methods such as STARR-seq.

### Pig enhancers are enriched in repetitive sequences

**Fig. 2:**
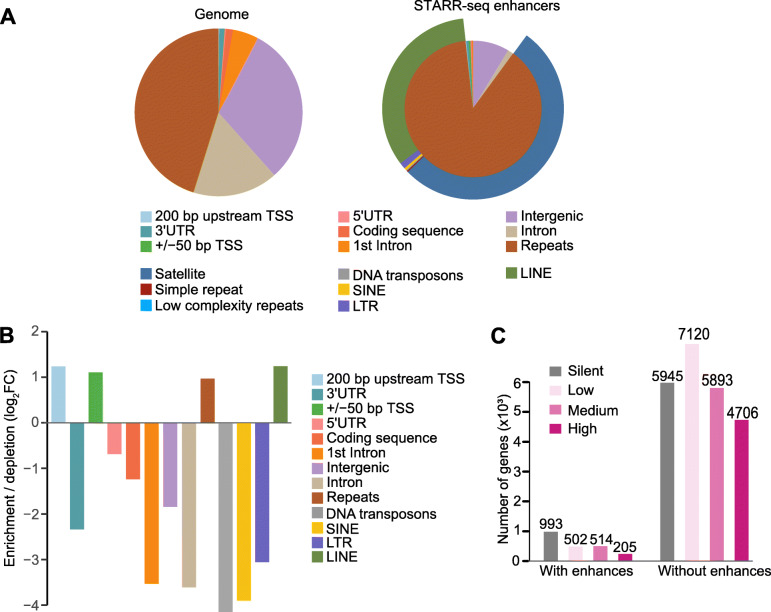
Distribution of functional enhancers in the pig genome. **A-B** Distribution **(A)** and relative enrichment **(B)** of functional enhancers in pig genomic regions. FC, fold change. **C** Number of genes expressed at different levels. Genes were classified with or without enhancers, which is based on whether an enhancer was in its proximity. Genes are classified into four groups according to their expression level. Silent, FPKM = 0; low, 0 < FPKM ≤1; medium, 1 < FPKM ≤10; high, FPKM > 10

To reveal the distribution pattern of enhancers in the pig genome, we annotated and calculated the percentage of enhancers in different functional genomic regions. The majority (2272/2576 = 88.19%) of the enhancers were located in repetitive sequences (Sscrofa11.1), and secondly in intergenic regions (222/2576 = 8.61%) (Fig. 2A and Additional file 5: Table S4). Previous studies indicated that transposable elements (TEs) are a widespread class of repetitive sequences in genomes [41, 42]. Thus, we analyzed the percentage of enhancers located in TE regions and found a high proportion of the enhancers were located in TEs (907/2576 = 35.20%), including short interspersed nuclear elements (SINEs) (29/2576 = 1.13%), long interspersed nuclear elements (LINEs) (863/2576 = 33.50%), long terminal repeats (LTRs) (11/2576 = 0.43%) and DNA transposons (4/2576 = 0.15%) (Fig. 2A). There is also a relative enrichment of the enhancers in LINEs compared to other TE classes, which was not due to LINEs abundance in the pig genome (Fig. 2B). Our observations are in line with previous reports of STARR-seq in human ESCs (Embryonic Stem Cells) [19], human HeLa-S3 cells [18], mouse ESCs [23] and rice [21]. These results indicate that certain families of TEs contribute to enhancer function [19].

Additionally, enhancers were overrepresented in the proximity of transcription start sites (TSSs) (Fig. 2B), emphasizing the importance of these regions for transcriptional regulation. This observation is consistent with the enhancers identified in *Drosophila* [15]. We also found that the pig enhancers were underrepresented in 1st intron, 5'UTR and 3'UTR (Fig. 2B), which is strikingly different with previous studies [15, 17, 21], although this could be a species-specific difference in enhancer distribution.

### Enhancers are enriched with silent and lowly expressed genes

To investigate whether enhancers are preferentially enriched in active or silent genes, we separated genes into four groups based on gene expression levels (silent: FPKM = 0; low: 0 < FPKM ≤1; medium: 1 < FPKM ≤10; and high: FPKM > 10) detected by RNA-seq (Additional file 4: Table S3) and assigned each enhancer to a putative gene based on closest genomic distance. A total of 2214 genes were assigned as targets for these enhancers (Fig. 2C). Enhancers were preferentially enriched in silent and lowly expressed genes (Fig. 2C), in line with a previous report in rice [21] and indicating that STARR-seq enhancers are not necessarily enriched within actively transcribed genes in vivo [15, 21].

### The potential biological function of enhancers

Assigning an enhancer to its target gene is challenging. For genes that were located close to enhancers, we further examined if these genes were enriched with specific biological functions. GO analysis for the closest genes of enhancers found that they were strikingly enriched in the cell projection morphogenesis, gene transcription and positive regulation of developmental biological processes (Additional file 3: Fig. S9). Furthermore, the enhancers were enriched in motifs of transcription factor (TF) binding sites, including Myod1, Tcfap2c and Zbtb3 (Additional file 6: Table S5). Myod1 is required for myogenin activation and affects the terminal differentiation of muscle cells [43], which indicated that the enhancers may participate in muscle development in the pig.

### Pig enhancers poorly overlap with accessible chromatin regions

To examine the enhancers with endogenous chromatin accessibility, we performed ATAC-seq in the PK15 cells (Additional file 4: Table S3) and found that 19.41% (500/2576) of the enhancers were accessible (Additional file 3: Fig. S10A and Additional file 7: Table S6). The low chromatin accessibility of the STARR-seq enhancers is also reported in previous studies [17, 21, 23]. Additionally, the enrichment of accessible chromatin regions was poorly correlated with STARR-seq enhancer strength (Additional file 3: Fig. S10B), indicating that pig enhancer strength cannot easily be predicted from chromatin accessibility features. From the above, this indicates that enhancers predicted based on chromatin accessibility may not be reliable in the pig genome.

### Enhancers correlate with active and repressive chromatin states

**Fig. 3:**
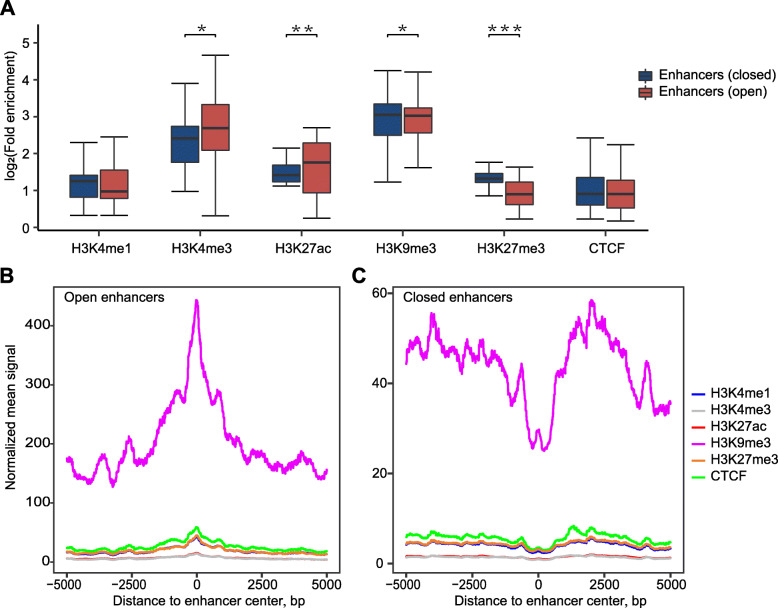
Enhancers correlate with both active and repressive chromatin states. **A** Comparison of the fold enrichment of epigenetic mark signals between open (red) and closed enhancers (blue). The open and closed enhancers were divided by ATAC-seq. (***P ≤ 0.001, ** *P* ≤ 0.05, * *P* ≤ 0.1, Wilcoxon rank-sum test). **B-C** Profiles of the enrichment signals of chromatin marks at open **(B)** and closed enhancers regions **(C)**. Normalized mean signal was calculated as the fold enrichment of the ChIP signal to the INPUT signal across 100 bp bins

We divided the pig enhancers into open enhancers, which overlap with ATAC-seq accessible chromatin regions, and closed enhancers, which did not locate in accessible chromatin regions, as defined in previous studies (Additional file 3: Fig. S10A and Additional file 7: Table S6) [15, 17, 21]. To compare the epigenetic marks between the open and closed enhancers, we performed ChIP-seq in PK15 cells (Additional file 4: Table S3) and integrated an analysis between the enhancer and ChIP-seq data (Fig. 3A-C and Additional file 7: Table S6).

Both the open and closed enhancers correlated with active and repressive chromatin states (Fig. 3A-C and Additional file 7: Table S6). Moreover, the open enhancers showed a higher fold enrichment compared with closed enhancers in active histone modification H3K4me3 (*P = 0.076, Wilcoxon rank-sum test) and H3K27ac (**P = 0.047, Wilcoxon rank-sum test) (Fig. 3A). In contrast, closed enhancers showed a significantly higher fold enrichment compared with open enhancers in repressive chromatin marks H3K9me3 (*P = 0.056, Wilcoxon rank-sum test) and H3K27me3 (***P = 3.8e-14, Wilcoxon rank-sum test) (Fig. 3A). Both closed and open enhancers displayed similar enrichment in H3K4me1 and insulator protein CTCF (Fig. 3A).

We found a subset of enhancers (133/2576 = 5.16%) that were enriched in active modifications and not in repressive modifications (Additional file 3: Fig. S11A and Additional file 7: Table S6). In addition, we also found a subset of enhancers (743/2576 = 28.84%) that were contrarily enriched in repressive modifications and not active modifications (Additional file 3: Fig. S11B and Additional file 7: Table S6). Interesting, a subset of enhancers (520/2576 = 20.19%) that correlated with active chromatin modifications was also enriched with repressive chromatin modifications (Additional file 3: Fig. S11C and Additional file 7: Table S6). These results indicate that enhancers correlated with both active and repressive chromatin modifications (Additional file 3: Fig. S11A-C). The apparently conflicting combination (Additional file 3: Fig. S11C) was also reported in rice [21] and it is assumed that enhancers were modified differentially in different subpopulations of the cells. Further studies are needed to test this hypothesis in different cell subpopulations.

### Most pig enhancers are endogenously repressed at the chromatin level

Additional, 43.83% (1129/2576) of the enhancers were enriched with the repressive histone signal H3K9me3 (Additional file 7: Table S6). Profiles along ±5-kb regions flanking the enhancers exhibited significantly higher signals for repressive modifications such as H3K9me3, CTCF and H3K27me3 than for active histone modifications including H3K4me1, H3K4me3 and H3K27ac in both open and closed enhancers (Fig. 3B and C). This result is consistent with majority of pig enhancers being located in repetitive sequences (2272/2576 = 88.19%) (Fig. 2A and Additional file 5: Table S4) and with repetitive sequences subjected to repressive epigenetic modifications [44–46]. From the above, we assume that most pig enhancers are endogenously repressed at the chromatin level and may function during cell type-specific development or at different developmental stages.

### Map enhancers to pig complex traits

Quantitative trait locus (QTL) and genome wide association study (GWAS) mapping for important economical traits have been widely applied in pigs [47–49]. Due to a large proportion of causal mutations being located in non-coding regions and a lack of annotation for non-coding regulatory elements in the pig, it is difficult to identify causal mutations for QTL and GWAS regions. In this study, we first annotated 2576 functional enhancers in the pig genome (Additional file 5: Table S4). We then integrated Hi-C data [38, 39] and QTL datasets [47] to identify enhancers that have biological associations with complex traits in the pig.

**Fig. 4:**
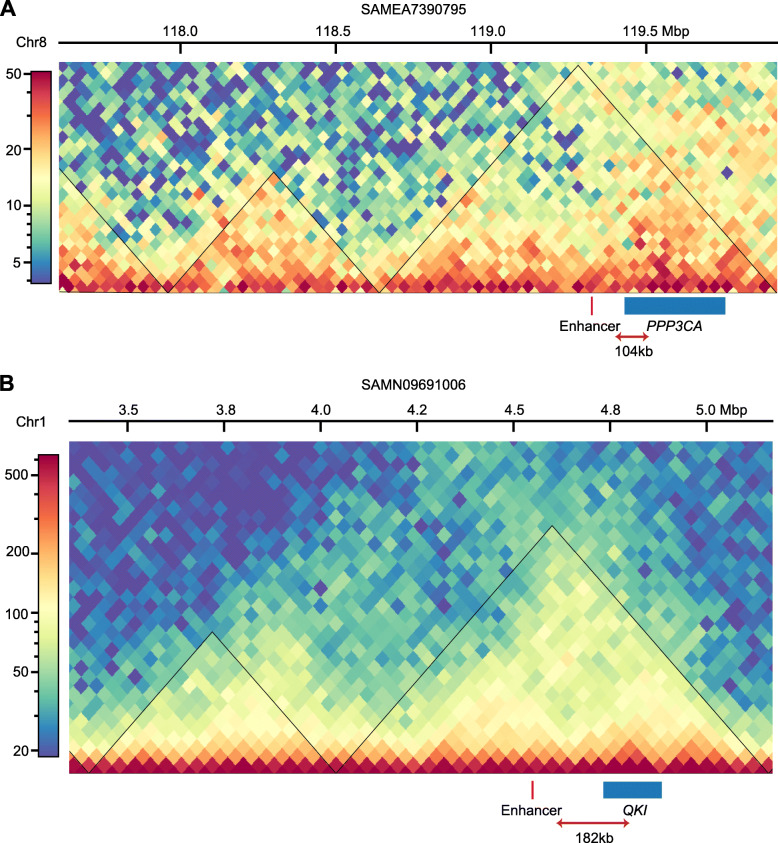
3D structure of an enhancer that possibly regulate protein-coding genes related to complex traits in the pig. **A** Hi-C contact heatmap of the chromosome 8 region shows than an observed functional enhancer (red box) and the *PPP3CA* gene (blue box) are in a same TAD (black triangles). Hi-C contact matrixes were built at 40 Kb resolution and used normalized reads from muscle tissue. **B** Hi-C contact heatmap of chromosome 1 region shows an observed a functional enhancer (red box) and the *QKI* gene (blue box) within a TAD (black triangles). Hi-C contact matrixes were built at 40 Kb resolution and used normalized reads from liver tissue

We deleted QTLs regions [47] that are longer than half a chromosome. And we analyzed filtered QTL regions that overlap with functional enhancer regions and found that 1508 non-redundant QTL regions associate with 440 traits enriched with enhancers (Additional file 8: Table S7). Among the identified regions, Lee et al. [50] reported a 1 Mb QTL region on *Sus scrofa* chromosome 8 that may impact the loin muscle area trait and contained the candidate gene *PPP3CA*. The QTL region overlaps with a functional pig enhancer (Sscrofa11.1, 8:119,324,324-119,325,095) (Additional file 8: Table S7). Previous studies reported that *PPP3CA* (also called calcineurin Aα) is actively involved in regulating the muscle fiber phenotype in pig [51, 52]. By reanalyzing the Hi-C data performed in pig fetal muscle tissues at 90 and 110 days of gestation [38], we found that the enhancer and *PPP3CA* gene were encompassed by a topologically associating domain (TAD) and linked by significant Hi-C contact, which indicates that the *PPP3CA* gene is associated with the loin muscle area trait in the pig and is potentially regulated by a distal functional enhancer (Sscrofa11.1, 8:119,324,324-119,325,095) (Fig. 4A and Additional file 3: Fig. S12). Further experimental investigations are required to validate the regulatory relationship between *PPP3CA* and the functional enhancer in pig muscle tissue.

Additionally, Bovo et al. [53] reported a 0.42 Mb QTL region on *Sus scrofa* chromosome 1 that showed effects on alkaline phosphatase (ALP) levels in the pig. The QTL region contains a functional enhancer (Sscrofa11.1, 1:4,548,557-4,549,774), which is 182 Kb upstream of the KH domain containing RNA binding (*QKI*) gene (Fig. 4B). A previous study showed that *QKI* regulates the expression of ALP and functions as critical regulator for colon epithelial differentiation [54]. Moreover, ALP isoenzymes are derived from the bones and liver, which are of particular interest for the evaluation of disease state [53, 55]. By reanalyzing the Hi-C data of female pig adult and fetus liver tissue [39], we found that the enhancer and *QKI* gene are connected by chromatin interaction within a TAD (Fig. 4B and Additional file 3: Fig. S13). From the above, we assume that a distal functional enhancer (Sscrofa11.1, 1:4,548,557-4,549,774) regulates the expression level of *QKI* and thus, influences ALP activity levels. The influence of the causal mutations in the enhancer region for ALP activity needs further functional validation.

## Discussion

This study is the first genome-wide identification of functional enhancers in the pig. We identified 2576 functional enhancers in the pig genome and revealed several different features of these functional enhancers compared with other species (Figs. 2 and 3 and Additional file 5: Table S4). Specifically, the number of pig functional enhancers detected in this study was less than seen in the human genome [17]. This might be due to our highly stringent selection criteria that included removing PCR replicates and a *q*-value cutoff to identify functional enhancers with greater confidence. Moreover, applying STARR-seq to the pig genome is still very challenging, due to the large size and complexity of this genome. Thus, new methods are needed for future studies on the detection of functional enhancers from highly complex mammalian genomes.

Previous studies used sequence homology information [11] and chromatin features, including DNA accessibility and active histone modifications, to identify potential enhancers [12–14]. Enhancer function in cell type-specific and developmental-stage-specific stage [56–58]. For this reason, methods based on DNA sequences or chromatin states can lead to many false positive predictions of enhancers that might not play functional roles in cells [15, 59]. Furthermore, these methods can only identify putative enhancers based on chromatin features or sequences and therefore do not exhaustively detect functional enhancers genome-wide. Thus, enhancers identified by chromatin features or sequences should be confirmed using approaches such as STARR-seq or by a RT-qPCR method. In our study, we found that most functional enhancers poorly overlap with chromatin accessible regions (Additional file 3: Fig. S10A) and are highly enriched with the repressive histone modification H3K9me3 (Fig. 3B and C). These results were consistent with previous studies showing that STARR-seq can identify some closed chromatin functional enhancers [15, 17, 23] and those functional enhancers can be silent or active, depending on the cell type and the developmental stage. As such, identification and characterization of pig functional enhancers under various cell types, including embryonic stem cells and primary cell cultures, in the future will be important for understanding the genetic basis of development. Moreover, a previous study assessed the effects of cis and trans for regulatory elements in human and mouse [60]. Future studies are needed to analyze and compare the cis and trans effects of regulatory elements such as enhancers and promoters between pigs and other mammalian species.

Previous studies have indicated that strong selection signals and regions associated with complex traits in the pig genome are located in non-coding regions [9, 61–63]. Here, we analyzed the functional enhancers that have the potential to regulate complex traits by integrating Hi-C and QTL data. This analysis identified two examples of enhancers that likely have distal regulatory function: (1) one enhancer may regulate the expression of the *PPP3CA* gene and thus, influence the loin muscle area trait, and (2) a second enhancer may regulate the *QKI* gene associated with ALP activity levels. *PPP3CA* is regarded as a candidate gene that plays an important role in muscle fiber differentiation and affects meat quality of livestock [64]. The *QKI* gene has been identified as the culprit gene for a patient with intellectual disabilities and has important roles in broader biological systems, such as cardiovascular development, bone metabolism and cancer progression [65, 66]. Additional studies are needed to refine the causal mutations in the enhancers and clarify the role of *PPP3CA* and *QKI* genes in muscle development and physiological processes in the pigs, respectively. The comprehensively identified pig functional enhancers found in this study provide insights into the functional complexity of enhancers in the pig.

## Conclusions

In all, we performed STARR-seq in pig PK15 and ST cells and identified 2576 functional enhancers in the pig genome. These enhancers poorly overlap with chromatin accessible regions and are highly enriched with the repressive histone modification H3K9me3. By integrating functional enhancers with pig complex traits, we found that the *PPP3CA* gene is associated with the loin muscle area trait and the *QKI* gene is associated with ALP activity levels, with both genes regulated by distal functional enhancers. Our first map of functional enhancers in the pig genome provide an important resource for enhancer studies and supply the new regulatory elements for pig breeding and construction of human disease models.

## Supplementary Information

**Additional file 1: Table S1.** Enhancers verified using the RT-qPCR method. Primers used to construct the reporter vectors.

**Additional file 2: Table S2.** Primers used for RT-qPCR.

**Additional file 3: Fig. S1.** DNA transfection induced immunoreaction and its suppression with treatment with immune inhibitors in ST cells. **Fig. S2.** Assessment of the immunoreaction (DMSO) and treatment effect of immune inhibitors after DNA is transfected into cells. **Fig. S3.** Distribution of fragment sizes in the STARR-seq libraries. **Fig. S4.** Genome coverage of STARR-seq libraries in pig non-repetitive regions. **Fig. S5.** GC-content analysis for the STARR-seq libraries. **Fig. S6.** Reproducibility of STARR-seq. **Fig. S7.** Correlation analysis of enhancers strength in two biological replicates of ST cells. **Fig. S8.** Venn diagram showing the overlap of enhancers between our STARR-seq approach and other published studies. **Fig. S9.** GO analysis of genes in proximity to enhancers. **Fig. S10.** ATAC-seq and STARR-seq enhancers integrated analysis. **Fig. S11.** Snapshots of signals at three types of chromatin state for the STARR-seq enhancer regions. **Fig. S12.** The enhancer (Sscrofa11.1, 8:119,324,324-119,325,095) and *PPP3CA* gene interacted by a TAD region in pig muscle tissues. **Fig. S13.** The enhancer (Sscrofa11.1, 1:4,548,557-4,549,774) and *QKI* gene interacted by a TAD region in pig liver tissues.

**Additional file 4: Table S3.** Sequencing statistics of STARR-seq, RNA-seq, ATAC-seq and ChIP-seq libraries.

**Additional file 5: Table S4**. Characterization of a total of 2576 functional enhancers in the pig genome.

**Additional file 6: Table S5.** Potential transcription factor binding sites in the enhancers.

**Additional file 7: Table S6**. Epigenetic mark enrichment analysis of the pig enhancers.

**Additional file 8: Table S7.** QTL regions overlapping functional enhancers.

## Data Availability

The sequencing datasets generated in this study were submitted to the Genome Sequence Archive (GSA) with ID CRA006465.

## Abbreviations

ALP: Alkaline phosphatase
ESCs: Embryonic stem cells
GO: Gene Ontology
GWAS: Genome-wide association study
Hi-C: High-throughput chromosome conformation capture
LINEs: Long interspersed nuclear elements
LTRs: Long terminal repeats
OD: Optical density
QTL: Quantitative trait locus
SCP1: Super core promoter 1
Shh: Sonic hedgehog
SINEs: Short interspersed nuclear elements
STARR-seq: Self-transcribing active regulatory region sequencing
TAD: Topologically associating domain
TEs: Transposable elements
TF: Transcription factor
TSSs: Transcription start sites
