# Supplementary material for: Genome-wide identification of functional enhancers and their potential roles in pig breeding
Source: J Anim Sci Biotechnol. 2022 Jul 4;13:75. doi: 10.1186/s40104-022-00726-y (PMC9252078; doi:10.1186/s40104-022-00726-y)
Supplement: Supplementary file 3 — Additional file 3: Fig. S1. DNA transfection induced immunoreaction and its suppression with treatment with immune inhibitors in ST cells. Fig. S2. Assessment of the immunoreaction (DMSO) and treatment effect of immune inhibitors after DNA is transfected into cells. Fig. S3. Distribution of fragment sizes in the STARR-seq libraries. Fig. S4. Genome coverage of STARR-seq libraries in pig non-repetitive regions. Fig. S5. GC-content analysis for the STARR-seq libraries. Fig. S6. Reproducibility of STARR-seq. Fig. S7. Correlation analysis of enhancers strength in two biological replicates of ST cells. Fig. S8. Venn diagram showing the overlap of enhancers between our STARR-seq approach and other published studies. Fig. S9. GO analysis of genes in proximity to enhancers. Fig. S10. ATAC-seq and STARR-seq enhancers integrated analysis. Fig. S11. Snapshots of signals at three types of chromatin state for the STARR-seq enhancer regions. Fig. S12. The enhancer (Sscrofa11.1, 8:119,324,324-119,325,095) and PPP3CA gene interacted by a TAD region in pig muscle tissues. Fig. S13. The enhancer (Sscrofa11.1, 1:4,548,557-4,549,774) and QKI gene interacted by a TAD region in pig liver tissues. [file 40104_2022_726_MOESM3_ESM.docx]

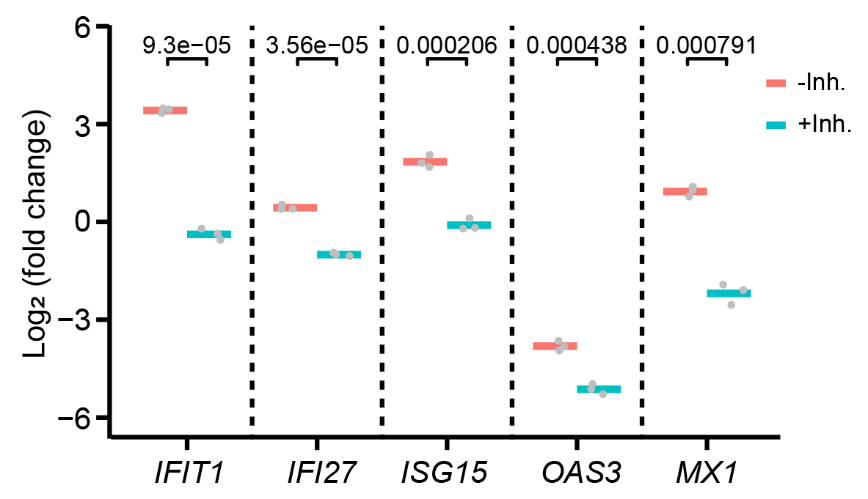


**Fig. S1.** DNA transfection induced immunoreaction and its suppression with treatment with immune inhibitors in ST cells. Assessment of immunoreaction and treatment with TBK1/IKK/PKR inhibitors after DNA is transfected into ST cells. Expression levels were assessed by RT-qPCR and normalized to non-transfected cells. Bars represent mean fold change across three independent replicates (grey dots). *P* values were calculated by *t*-test.


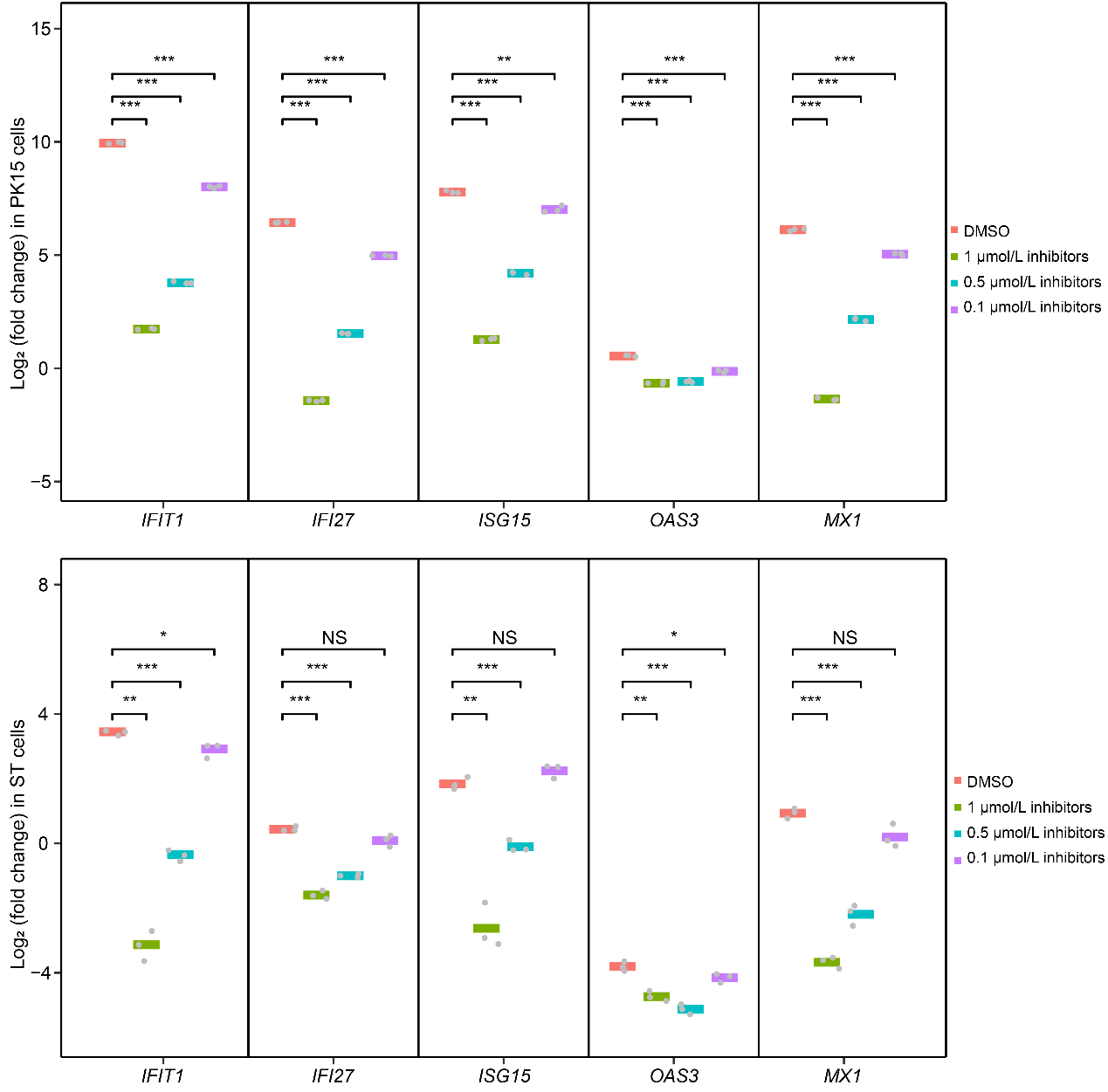


**Fig. S2.** Assessment of the immunoreaction (DMSO) and treatment effect of immune inhibitors after DNA is transfected into cells. **(A-B)** DNA transfection induced immunoreaction (DMSO) and treated with immune inhibitors in PK15 **(A)** and ST cells **(B)**. The expression levels were assessed by RT-qPCR and normalized to non-transfected cells. Error bars represent three independent replicates.


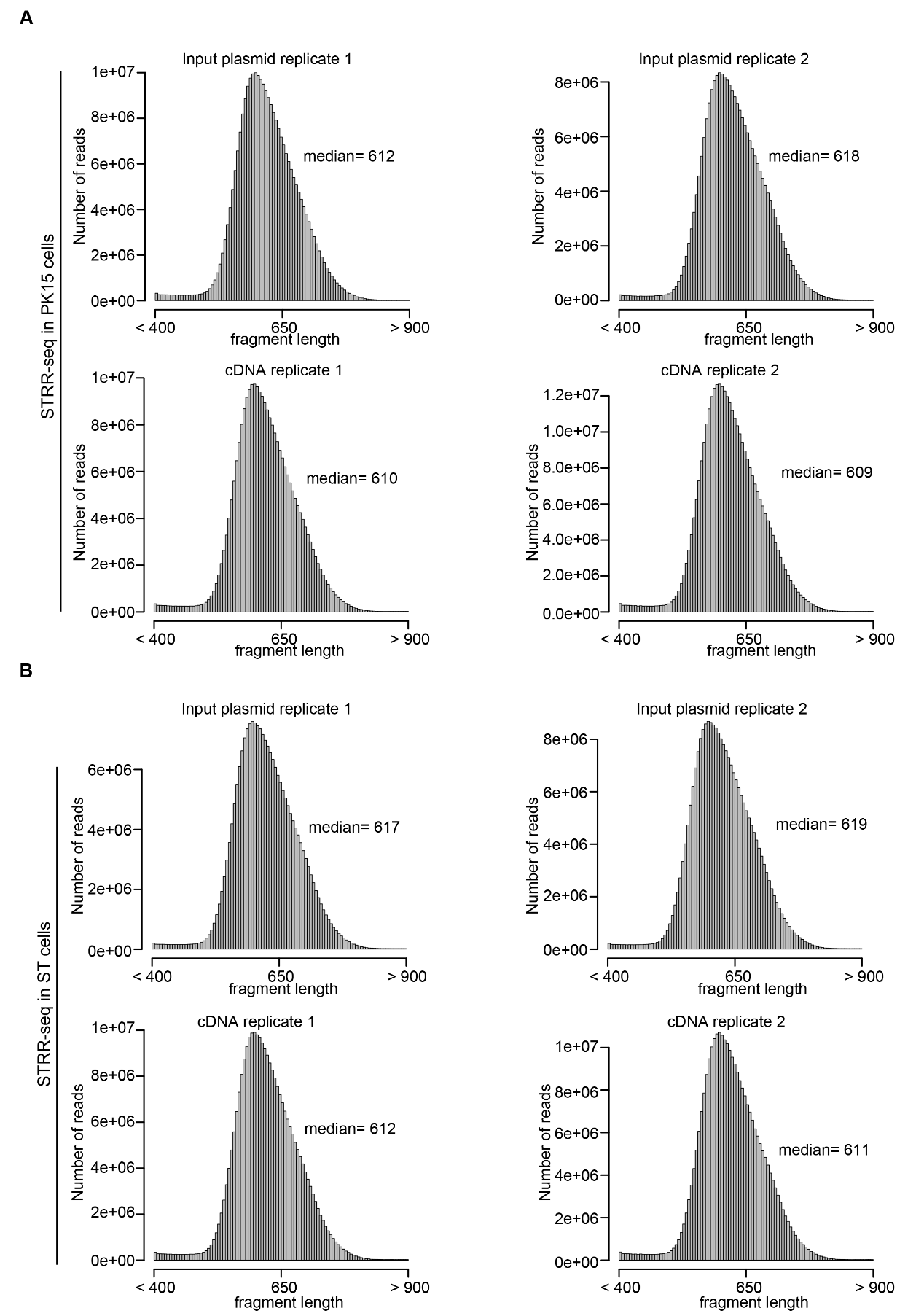


**Fig. S3.** Distribution of fragment sizes in the STARR-seq libraries. **(A-B)** Distribution of fragment sizes in the STARR-seq input plasmid and the cDNA libraries for PK15 **(A)** and ST cells **(B)**, with the median fragment sizes indicated.


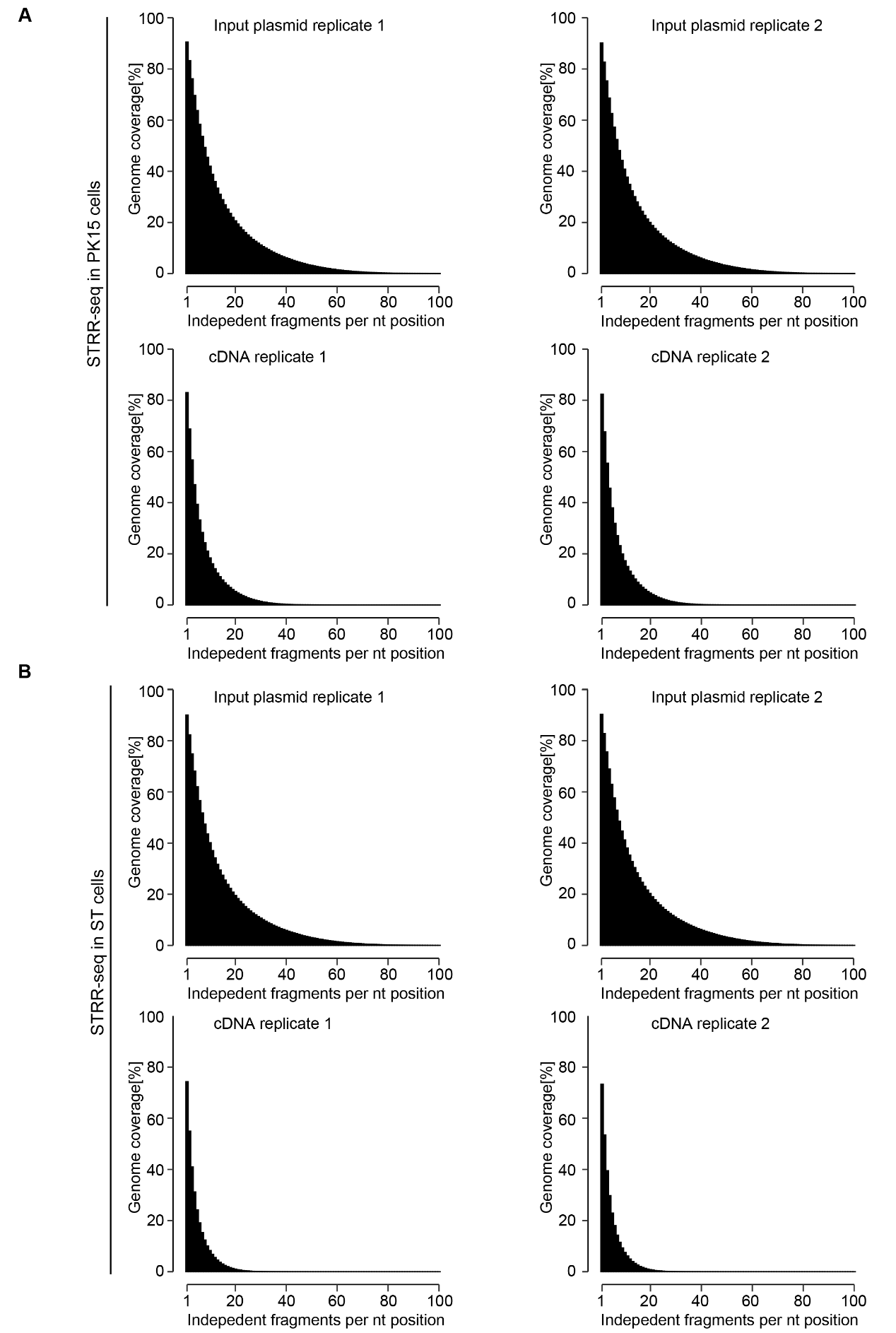


**Fig. S4.** Genome coverage of STARR-seq libraries in pig non-repetitive regions. **(A-B)** Coverage of the pig non-repetitive genomic regions using independent fragments (cumulative) for the STARR-seq libraries in PK15 **(A)** and ST **(B)**.


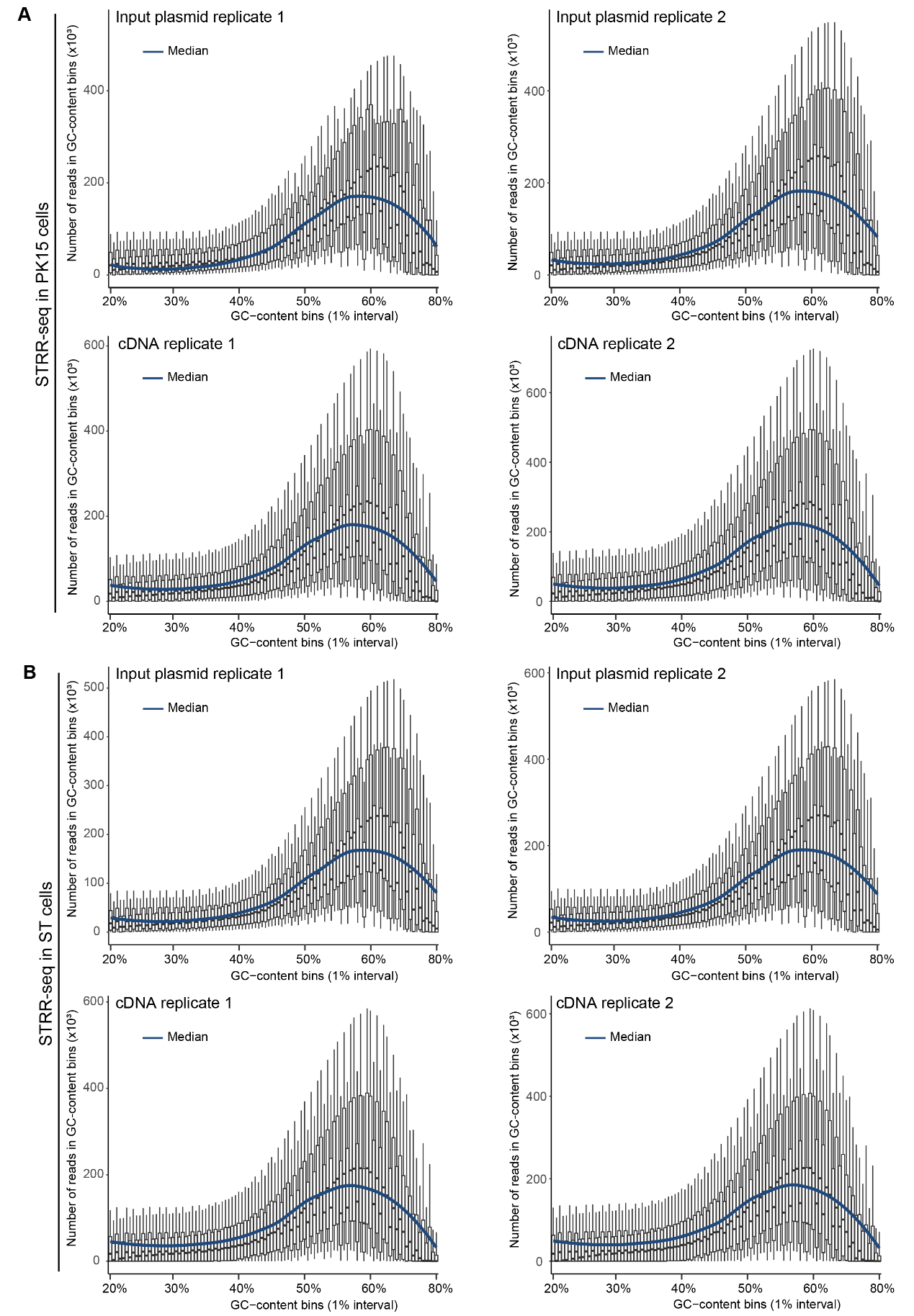


**Fig. S5.** GC-content analysis for the STARR-seq libraries. **(A-B)** The pig non-repetitive genome was divided into 100 bp windows and binned together according to GC-content. Each boxplot shows the read depth of all positions within the respective windows for the STARR-seq libraries in PK15 **(A)** and ST cells **(B)**. For each GC-content bin, if a genomic window was covered by more fragments than 50% of genomic windows of the same GC-content, then the number of fragments covering this genomic window was set as the median value.


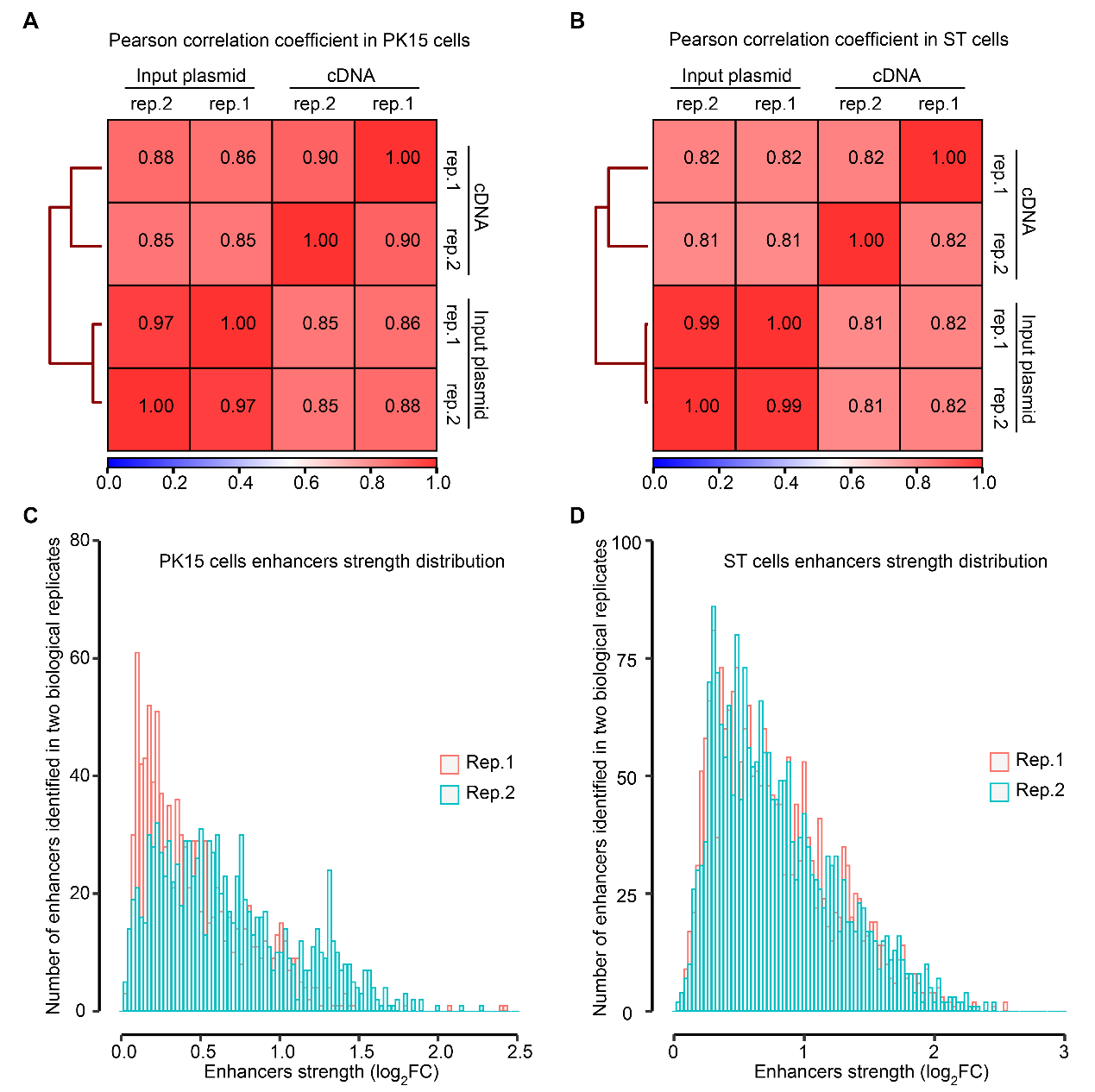


**Fig. S6.** Reproducibility of STARR-seq. **(A-B)** Correlation analysis of sequenced libraries in PK15 **(A)** and ST cells **(B)**. The pig genome was divided into 1000 bp bins and read coverage was calculated for each bin. Correlation values for read coverage between the sequenced libraries were calculated using the Pearson’s Correlation Coefficient. **(C-D)** Distribution of STARR-seq strength values in two biological replicates of PK15 **(C)** and ST cells **(D)**. Enhancer strength was calculated based on fold change (FC, cDNA reads count divided by input plasmid reads number) using 600 bp windows along each chromosome.

**
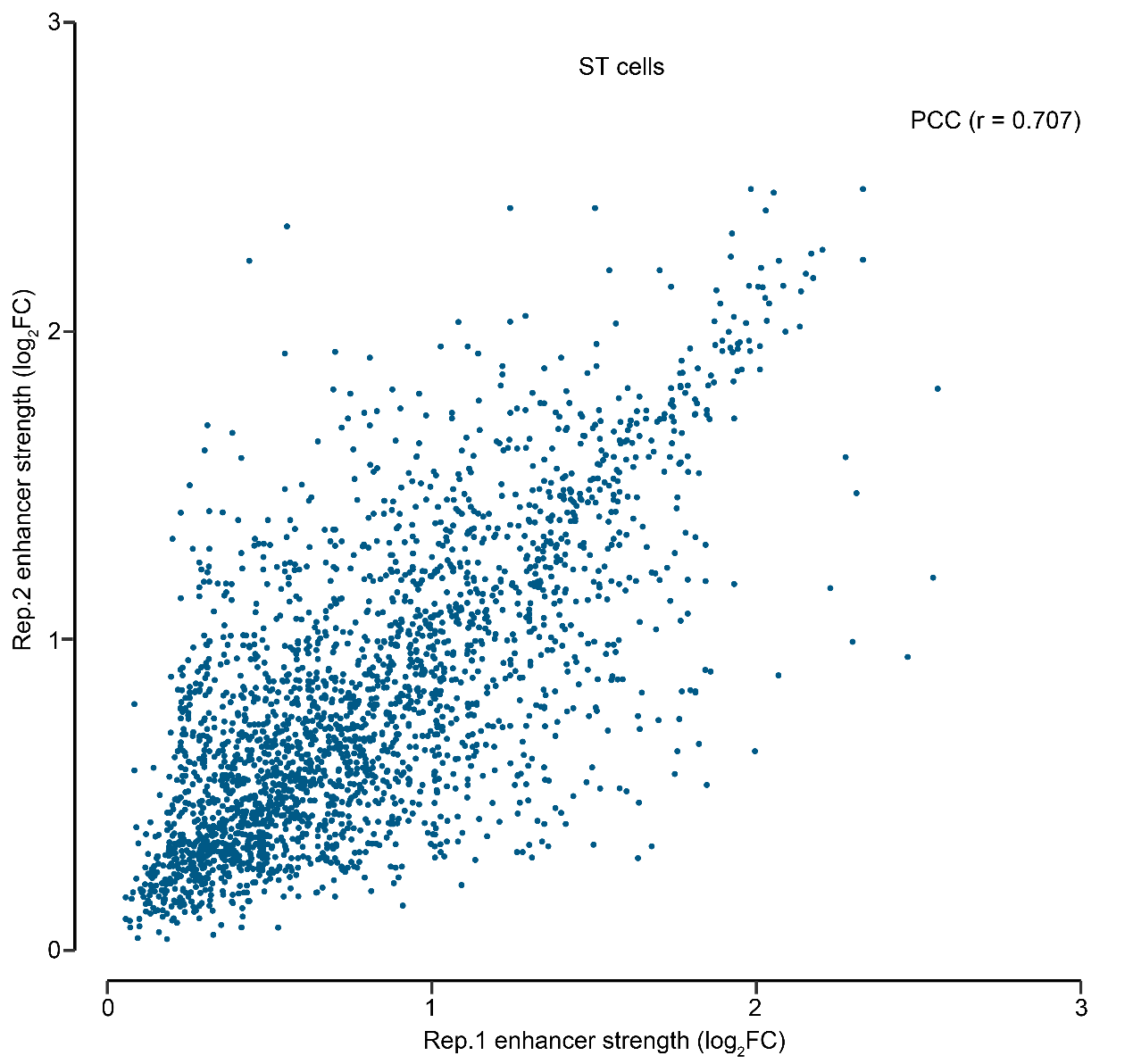
**

**Fig. S7.** Correlation analysis of enhancers strength in two biological replicates of ST cells. The correlation was evaluated using Pearson’s Correlation Coefficient (PCC). Enhancer strength was calculated based on fold change (FC, cDNA reads count divided by input plasmid reads number) using 600 bp windows along each chromosome.


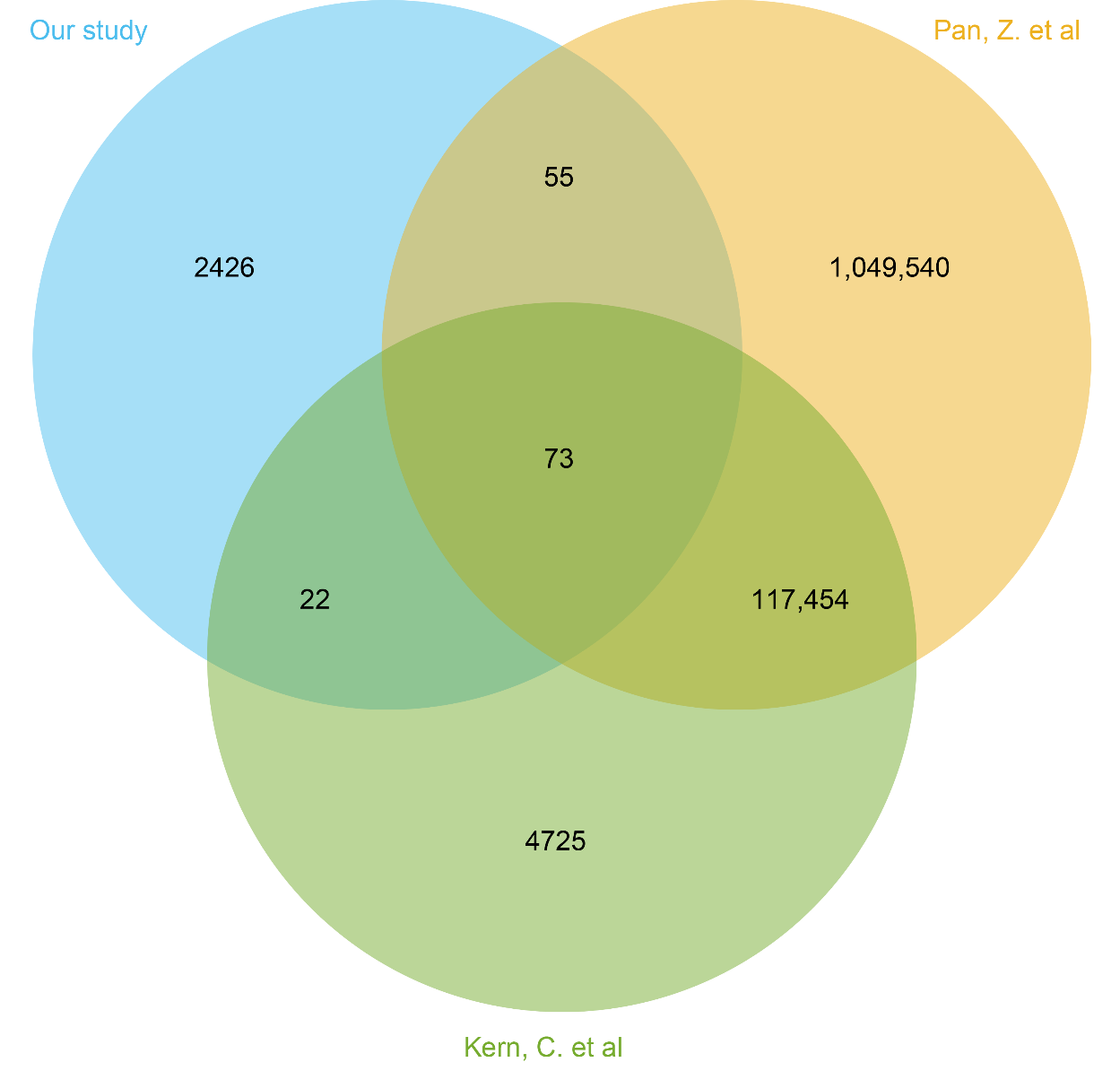


**Fig. S8.** Venn diagram showing the overlap of enhancers between our STARR-seq approach and other published studies. Numbers indicated statistics of enhancers identified by STARR-seq approach and enhancers predicted by ChIP-seq using chromatin marks from Pan et al. [15]. and Kern et al. [13].


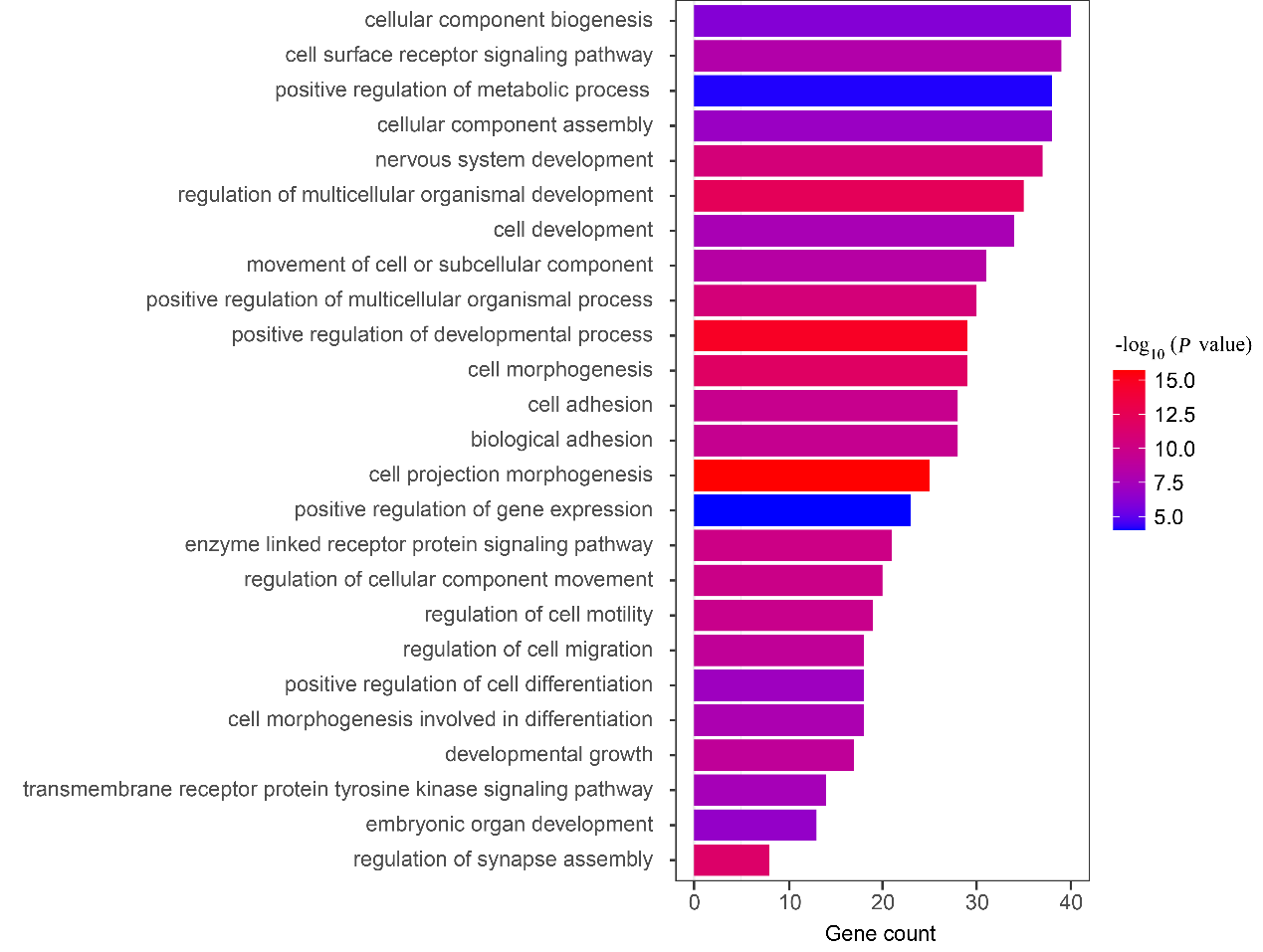


**Fig. S9.** GO analysis of genes in proximity to enhancers. Each enhancer was assigned to a putative gene based on closest genomic distance. The top 25 categories of GO terms are shown.


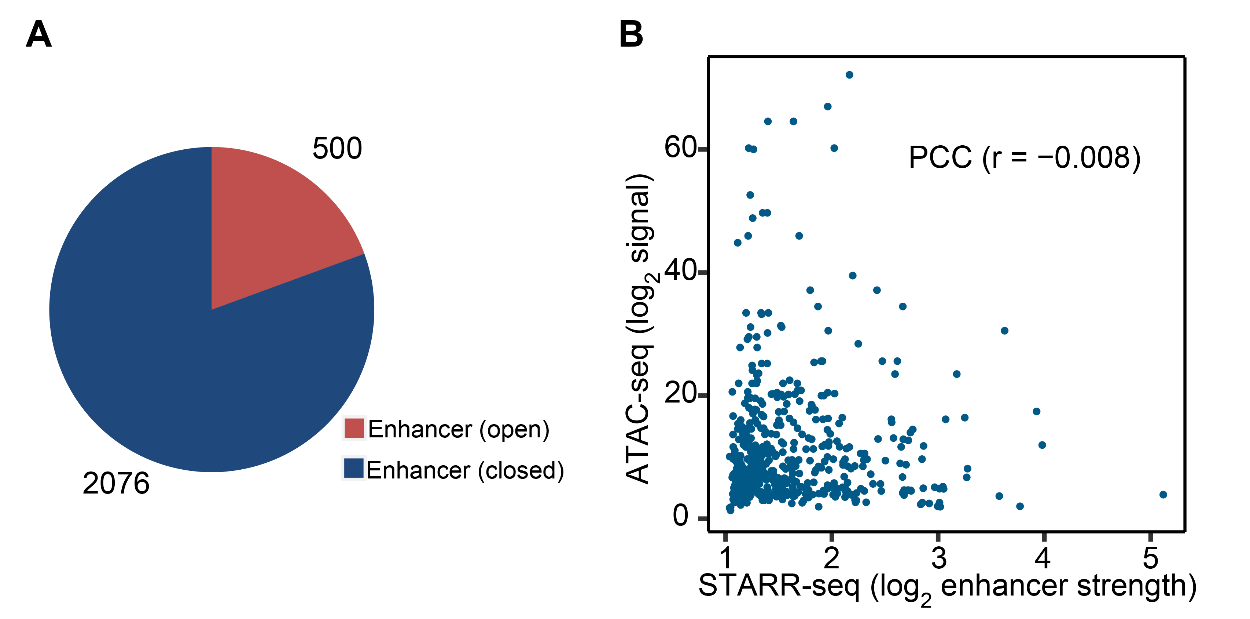


**Fig. S10.** ATAC-seq and STARR-seq enhancers integrated analysis. **(A)** Statistics of the open (red) and closed enhancers (blue). The open and closed enhancers were divided by ATAC-seq. **(B)** Correlation analysis of STARR-seq enhancers strength and ATAC-seq signal. PCC, Pearson’s Correlation Coefficient (r = –0.008). Enhancer strength was calculated based on fold change (FC, cDNA reads count divided by input plasmid reads number) using 600 bp windows along each chromosome.


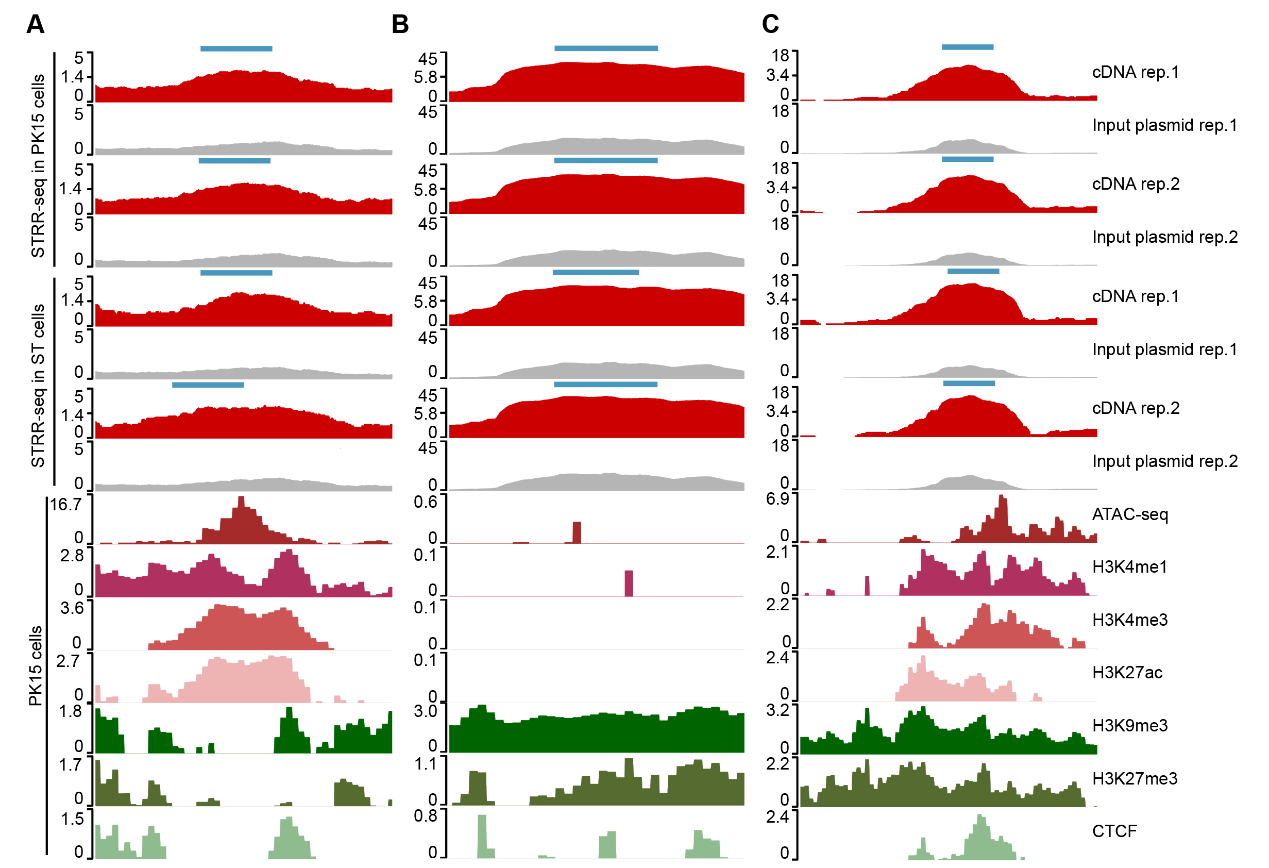


**Fig. S11.** Snapshots of signals at three types of chromatin state for the STARR-seq enhancer regions. **(A-C)** Enhancers located in active (ATAC-seq, H3K4me1, H3K4me3 and H3K27ac) (**A)**, repressive (H3K9me3, H3K27me3 and CTCF) **(B)**, and both types of chromatin state **(C)** regions. Blue boxes denote the identified STARR-seq enhancers in PK15 and ST cells. Signal intensities of ATAC-seq and ChIP-seq tracks are shown as RPKM. ChIP-seq tracks depict the signal intensities of ChIP signal over INPUT signal.

**
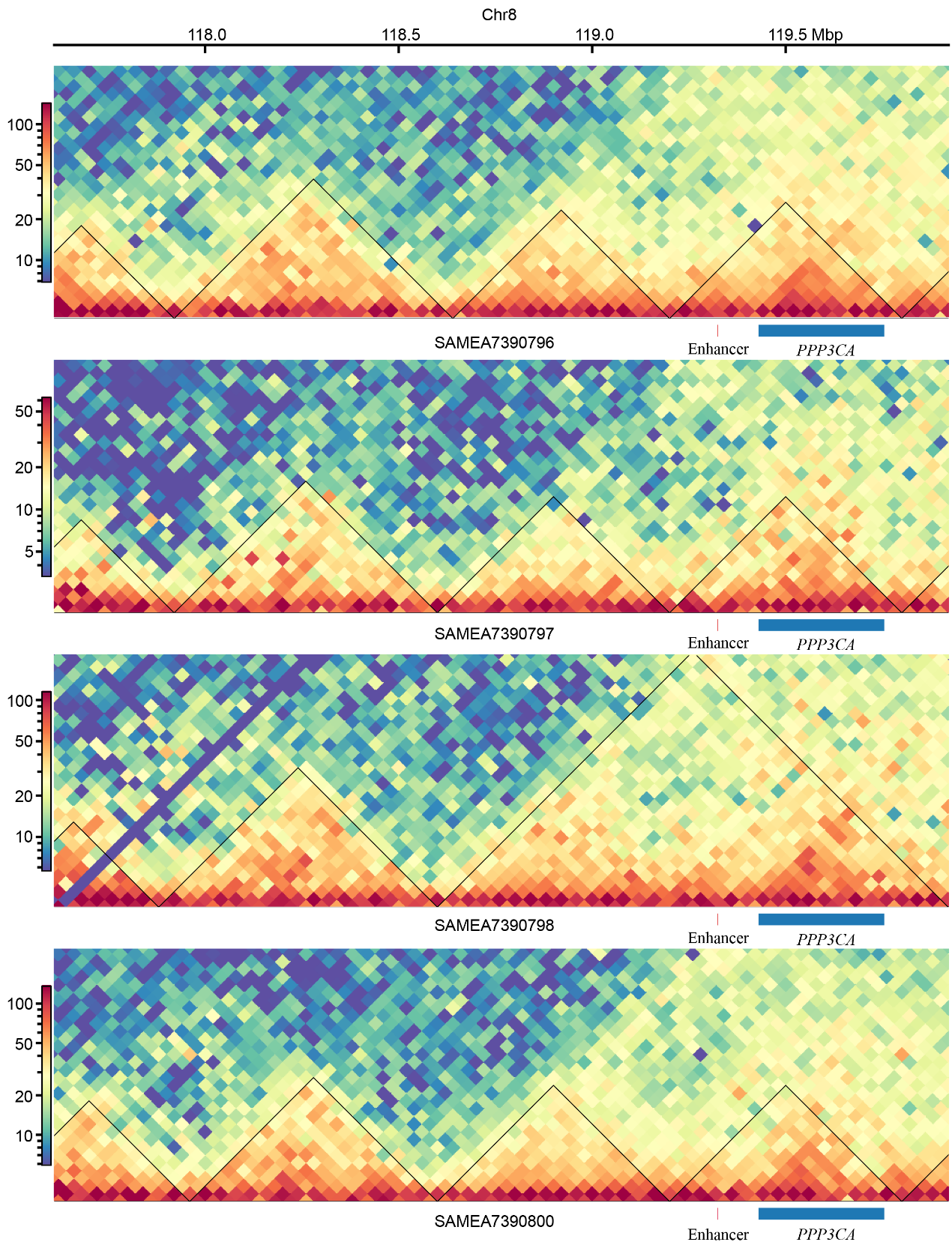
**

**Fig. S12.** The enhancer (Sscrofa11.1, 8:119,324,324-119,325,095) and *PPP3CA* gene interacted by a TAD region in pig muscle tissues. Hi-C contact heatmap of chromosome 8 region observed functional enhancer (red box) and *PPP3CA* gene (blue box) within a TAD (black triangles). The Hi-C contact matrixes were built at 40 Kb resolution and used normalized reads in muscle tissues.


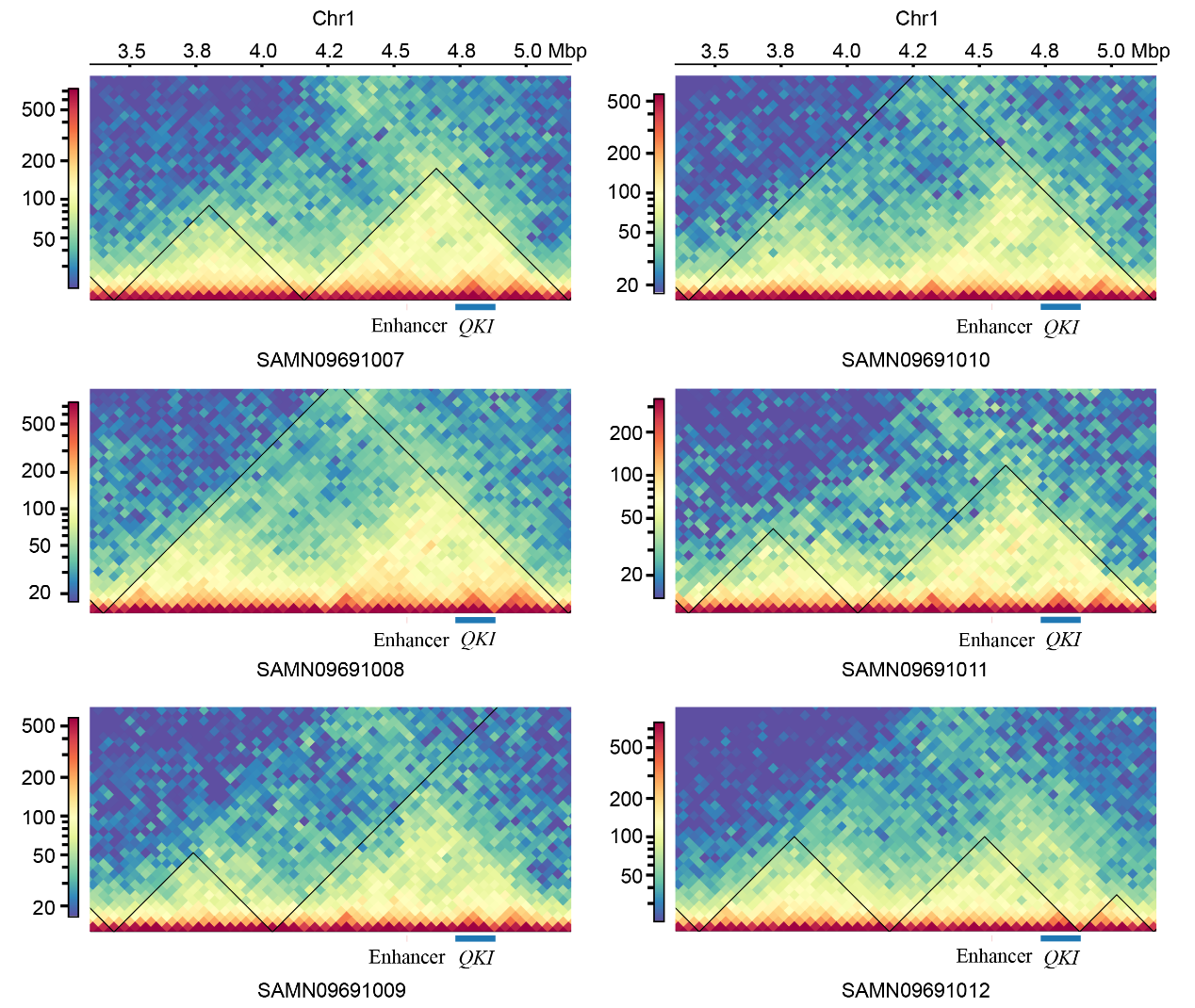


**Fig. S13.** The enhancer (Sscrofa11.1, 1:4,548,557-4,549,774) and *QKI* gene interacted by a TAD region in pig liver tissues. Hi-C contact heatmap of chromosome 1 region observed functional enhancer (red box) and *QKI* gene (blue box) within a TAD (black triangles). The Hi-C contact matrixes were built at 40 Kb resolution and used normalized reads in liver tissues.
